# Achieving self-sustainability of service delivery in an eye care program in Madagascar using time-driven activity based costing

**DOI:** 10.1186/s12913-020-05074-z

**Published:** 2020-03-12

**Authors:** Philippe Rakotondrajoa, Tantely Rakotomamonjy, Randrianaivo Jean Baptiste, Lisa Demers, Peter Kileo, Michele Anholt, Jaafar Aghajanian, Ken Bassett

**Affiliations:** 1 Sampan’asa Loterana Momba Ny Fahasalamana (SALFA) network, Ampandrozonana, 208 Sambava, Madagascar; 2 Centre Hospitalier Universitaire (CHU) Toamasina - Service d’Ophtalmologie, 501 Toamasina, Madagascar; 3 Seva Canada, 100 – 2000 West 12th Avenue, Vancouver, BC V6J 2G2 Canada; 4 Kilimanjaro Center for Community Ophthalmology, P.O. Box 2254, Moshi, Tanzania; 5 One Health, Faculty of Veterinary Medicine, University of Calgary, 3280 Hospital Drive NW, Calgary, AB T2N 4Z6 Canada; 6 Department of Ophthalmology and Visual Sciences, British Columbia Centre for Epidemiologic and International Ophthalmology, University of British Columbia, 2550 Willow Street, Vancouver, BC V5Z 3N9 Canada

**Keywords:** Financial self-sustainability, Eye care programs, Madagascar, Activity based costing

## Abstract

**Background:**

In the absence of adequate and reliable external funding, eye care programs in developing countries need a high level of financial self-sustainability for maintenance and growth. To cope with these cost pressures, an eye care program in Sava, Madagascar adopted a Time-Driven Activity Based Costing (TDABC) methodology to better manage the cost of, and to improve revenue associated with, their three principle activities: consultation visits, cataract operations, and sale of glasses.

**Methods:**

Direct (variable) and indirect (fixed) cost estimates and revenue sources were gathered by activity (consultation, cataract operation, sale of glasses) and location (hospital or outreach) and TDABC models were established. Estimates were made of the proportion of the ophthalmologist’s time (by far the scarcest and most expensive resource) dedicated to consultation, cataract operation, or sale of glasses. These proportions were used to attribute costs by activity. The hospital manager and medical director modified staff roles, program activities, and infrastructure investments to reduce costs and expand revenue sources by activity while monitoring activity specific efficiency and profit.

**Results:**

The TDABC model for patient consultations showed that they were time consuming for the ophthalmologist and only resulted in net profit for the institution if the ophthalmologist converted most cataract patients into accepting surgery and refractive error patients into purchasing glasses from the hospital optical shop. The TDABC model for cataract surgery showed the programs needed to reduce the cost of imported consumable surgical products, reduce operation time, and, most importantly, reduce the number of very costly surgical camps providing essentially free surgery. In addition the model pushed the hospital to train staff in marketing skills so that a higher proportion of cataract cases come directly to the hospital willing to pay for surgery. The TDABC model provided the optical shop manager, for the first time, data on both the cost of supplies (frames and lenses) and the price of glasses sold resulting in strategies to maximize profit through preferential product presentation and customer experience. The eye program in the Sava region in northern Madagascar improved its cost recovery from 68 to 102% through patient revenue.

**Conclusions:**

TDABC models helped the Sava eye care program develop more efficient service delivery and increase revenue in excess of steadily increasing costs.

## Background

Madagascar is among the poorest 20% of developing countries with most people living below the poverty line on US$1.21 per day; one-quarter of the average personal income in sub-Saharan Africa [1]. The Malagasy government cannot provide even basic healthcare services leaving 25% of the population without access to any form of primary healthcare. The average distance to a health facility is over ten kilometers [2]. Eye care services are a low priority due to competing demands for health care funding including very high maternal and neonatal mortality, endemic debilitating infectious diseases, and chronic malnutrition [3].

The Kilimanjaro Center for Community Ophthalmology (KCCO), a community ophthalmology training institution dedicated to reducing blindness in Africa, began providing ongoing support for management, training and program planning to the Sava eye unit (part of the Sampan’asa Loterana Momba Ny Fahasalamana (SALFA) network in the Malagasy Lutheran Church Health Department) in 2009. Sava has one ophthalmologist, one ophthalmic nurse and two regular nurses and serves a primarily rural setting that relies on subsistence farming and fishing. KCCO helped Sava develop a community outreach plan which is the basis of its Vision 2020 Program. The outreach program involved selecting underserved but accessible sites for community visits, pre-visit marketing, on-site diagnosis including refraction and medical treatment, as well as transportation of patients to the base hospital for surgery, primarily cataract. Due to the limited number of staff, the hospital eye unit closed for outreach days.

Sava was established, and primarily supported, by an international non-governmental eye care organization. In 2011 most of the funding from the international donor stopped and the local Lions Sight First Program significantly reduced support for surgical supplies (medicine and intra-ocular lenses). As a result, the eye program turned to financial self-sustainability models for its survival.

In 2013, Seva Canada began training the Sava eye unit manager in financial data collection, reporting and management. At the time, financial record keeping was limited to staff payroll, a ledger listing patient registration fees, and receipts for expenditures including supplies, building and vehicle maintenance. Financial data was not organized to assess the cost and revenue of program activities. For the eye program, the principle activities were: outpatient consultation, surgical procedures (primarily cataract) and sale of glasses.

Detailed cost estimates were developed and assigned as fixed or variable [4] (Table 1). Variable costs (such as intra-ocular lenses and medicine) were affected by the volume of work performed, while fixed expenses (such as electricity, internet, and bank charges) were not.

**Table 1:** Fixed and variable cost categories as a percentage of revenue

Accounts	2015 Balances	Percentage of Revenue
**Revenue:**		
Patient revenue	91,688	89.53%
Donation revenue	10,316	10.07%
Bank Revenue	412	0.40%
**Total Revenue**	102,415	100.00%
**COGS:**		
Medications COGS	17,495	17.08%
Glasses COGS	6773	6.61%
Outreach Costs	577	0.56%
Others	802	0.78%
**Total COGS**	25,647	25.04%
**Gross Margin**	76,768	74.96%
**General and Admin Expense:**		
Electricity	1704	1.66%
Fuel	924	0.90%
Supplies & materials	2632	2.57%
Repair and Maintenance	3714	3.63%
Outside personnel	118	0.11%
Transportation - base	218	0.21%
Communication, internet, courier	2025	1.98%
Travel	2381	2.32%
Bank charges/Bank interest	179	0.17%
Personel salary, training, clothing etc	38,027	37.13%
Entertainment, unaccounted etc	4054	3.96%
Depreciation	17,742	17.32%
**Total General and Admin Exp.**	73,717	71.98%
**Net Income (Loss)**	3051	2.98%
**Patient revenue/All expenses**		92%
**Patient revenue/all revenue**		90%

Modified Time-Driven Activity Based Costing (TDABC) [5] models were established suitable for general management of the three principle activities (consultation, cataract operation, sale of glasses). The TDABC model was used to determine the cost of each activity as patients flow through various hospital processes. The TDABC model used two basic parameters: 1) the unit cost of resource input (labour and non-labour costs) and 2) the time and quantity of resources required to perform a transaction or an activity.

The TDABC models provided an infrastructure for understanding cost drivers [6] and revenue sources at an early stage in developing financial systems in this institution. To determine cost drivers six steps were followed: 1) identify staff and their activities 2) determine salaries 3) estimate practical capacity 4) calculate cost per time unit 5) determine the required time units for each activity and, 6) calculate cost per transaction.

## Methods

**Fig. 1:**
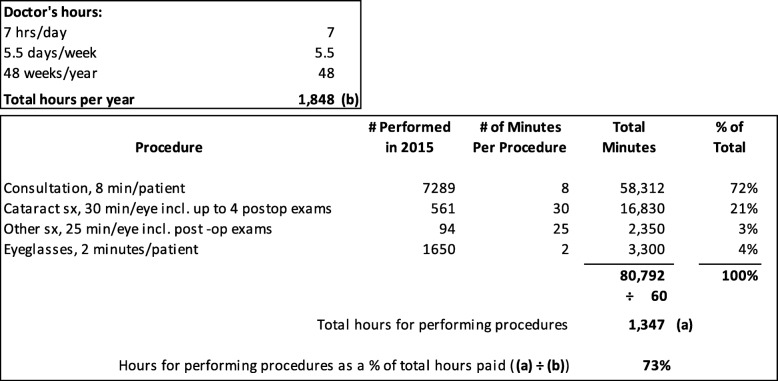
Calculation of ophthalmologist’s time spent on activities; Sava program data 2015

Fixed expenses were distributed to the 3 activities (consultation, cataract operations, sale of glasses) for the TDABC models. Specifically, all fixed costs were divided the same way, regardless of activity, according to the proportion of the ophthalmologist’s time spent on that activity. The proportion of the ophthalmologist’s time on each activity was estimated by dividing the total time the doctor worked per year by the total time spent on a given activity (time per activity times the total number of procedures per year) (Fig. 1).

Revenue was distributed in the TDABC model by activity and location (at the hospital, referred to as base, or at outreach) (Table 2). This enabled evaluation of revenue achieved in relation to the unit cost of an activity. The unit cost reflected the time spent on the activity by the ophthalmologist. As much as possible, each activity was considered to involve a distinct time period with no overlap [4].

**Table 2:** Cost and revenue distributed by activity

Description	Total Amount	Consultations		Cataract		Glasses	
		Base	Outreach	Base	Outreach	Base	Outreach
**Number of Procedures**		4724	2563	517	44	1119	531
**Direct/Variable Costs**							
Medications - Cost of goods sold	17,360	8567	2142	6130	522	–	–
Glasses - Cost of goods sold	6773	–	–	–	–	6129	644
Gas	78	–	–	59	–	20	–
Medication & spectacle deliveries	272	50	18	20	3	163	18
Outreach	577	–	373	–	91	–	112
Supplies	452	452	–	–	–	–	–
Total Direct Costs	25,512	9069	2533	6208	615	6312	774
**Indirect/Fixed Costs**							
Total Indirect Costs	71,587	34,499	18,717	14,155	1205	2042	969
**Total Costs**	97,100	43,568	21,251	20,363	1820	8355	1744
**Revenues**							
Consultations	8299	8299	–	–	–	–	–
Care and treatment	406	393	12	–	–	–	–
Hospitalization	128	55	–	72	–	–	–
Cataract Operations	22,331	–	–	22,331	–	–	–
Cataract Operations outreach	2020	–	–	–	2020	–	–
Prescription writing	25	25	–	–	–	–	–
Medication sales	22,316	22,316	–	–	–	–	–
Medication sales, outreach	5251	–	5251	–	–	–	–
Eyeglass sales, base	24,880	–	–	–	–	24,880	–
Eyeglass sales, outreach	1805	–	–	–	–	–	1805
Prosthesis	151	135	16	–	–	–	–
Eyeglass accessories	21	–	–	–	–	21	–
Others	48	48	–	–	–	–	–
**Total Revenues:**	87,680	31,271	5279	22,403	2020	24,901	1805
**Excess of Revenues over Expenses/(Expenses over Revenues)**	(9420)	(12,297)	(15,971)	2040	200	16,546	61
**Donations**							
Donations	16,294	3961	3721	2875	5391	235	111
Others revenues	400	193	104	79	7	11	5
**Total Donations/Other Revenue:**	16,694	4154	3826	2955	5397	246	117
**Excess of All Revenues over Expenses (Expenses over All Revenues)**	7274	(8143)	(12,145)	4995	5598	16,792	178

Using the average revenue per activity, average variable cost per activity and total fixed expenses, Sava was able to calculate the number of activities needed to reach 100% cost recovery. Sava set a goal of achieving at least 120% cost recovery of service delivery in order to replace aging ophthalmic equipment.

In addition to striving for time and cost efficiencies, the eye program recognized the need for, and value of, counselling by all clinical staff in order to increase the proportion of patients accepting cataract surgery and purchasing glasses at the hospital optical shop.

## Results

**Fig. 2:**
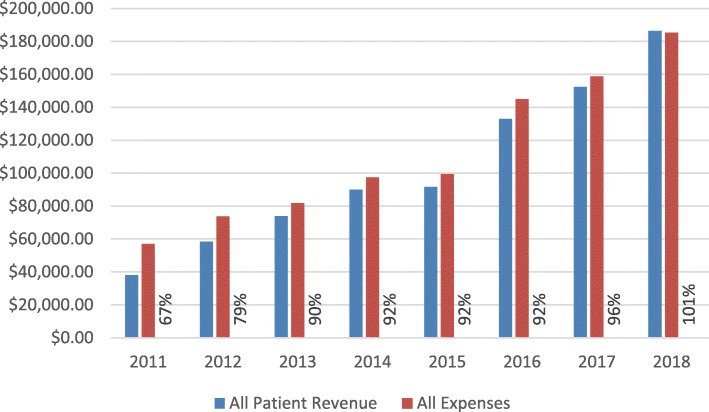
Total eye program revenue and expenses, by year

Sava gathered and reported data semi-annually by activity and location in a standardized Excel spread sheet from 2013 to 2018. Total costs, sales and volume were reported from 2013 to 2018 (Table 3). Total revenue and expenses were gathered from 2011 to 2018 (Fig. 2).

**Table 3:** Total costs, sales and volume, by activity and year

***Sava Eye Clinic***							
***Activity***	***Description***	***2013***	***2014***	***2015***	***2016***	***2017***	***2018***
*Consultations*	*Total Costs*	*$43,766*	*$55,998*	*$64,819*	*$93,256*	*$103,712*	*$114,381*
	*Total Sales*	*$23,167*	*$32,236*	*$36,550*	*$52,356*	*$65,487*	*$79,923*
	*% Cost Recovery*	*53%*	*58%*	*56%*	*56%*	*63%*	*70%*
	*Price per unit*	*2.95*	*$4.87*	*$5.02*	*$7.90*	*10.71*	*11.47*
	*Units*	*7852*	*6625*	*7287*	*6626*	*6113*	*6971*
*Glasses*	*Total Costs*	*$12,456*	*$12,460*	*$10,098*	*$18,250*	*$17,148*	*$29,998*
	*Total Sales*	*$23,475*	*$27,887*	*$26,706*	*$42,820*	*$44,569*	*$60,542*
	*% Cost Recovery*	*188%*	*224%*	*264%*	*235%*	*260%*	*202%*
	*Price per unit*	*11.93*	*$11.50*	*$16.19*	*$21.73*	*28.55*	*30.17*
	*Units*	*1968*	*2424*	*1650*	*1971*	*1561*	*2007*
*Cataract Operations*	*Total Costs*	*$24,420*	*$27,745*	*$22,183*	*$30,987*	*$33,726*	*$36,919*
	*Total Sales*	*$25,960*	*$28,493*	*$24,423*	*$32,353*	*$37,285*	*$39,857*
	*% Cost Recovery*	*106%*	*103%*	*110%*	*104%*	*111%*	*108%*
	*Price per unit*	*30.76*	*$37.25*	*$43.54*	*$58.50*	*69.05*	*68.13*
	*Units*	*844*	*765*	*561*	*553*	*540*	*585*
***Total***	***Total Costs***	***$81,851***	***$97,491***	***$99,379***	***$144,960***	***$158,798***	***$185,449***
	***Total Sales***	***$73,849***	***$90,087***	***$91,688***	***$132,951***	***$152,374***	***$186,459***
	***% Cost Recovery***	***90%***	***92%***	***92%***	***92%***	***96%***	***101%***

**Fig. 3:**
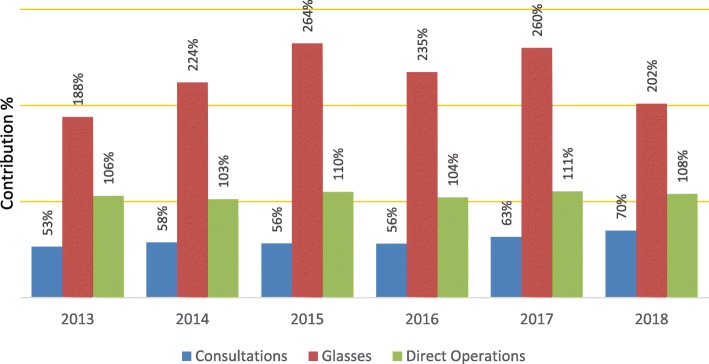
Proportion of cost recovery through revenue, by activity

By 2013 the revenue from the sale of glasses exceeded costs (Fig. 3). In 2018, glasses revenue covered 202% of costs. The programs increased the number of patients seen and productivity levels (patients seen per hour particularly by the ophthalmologist) without compromising the quality of care. In 2018, consultation fees recovered 70% of their activity costs (Fig. 3). Concurrently, the ophthalmologist recognized the need to improve his marketing skills to convince more patients to purchase glasses in the hospital optical shops. Sava reached the cataract breakeven point every year from 2013 to 2018. The revenue received from patients who paid for their cataract operations (direct patients) enabled an affordable fee for those too poor to pay full price. The result was increased revenue with the same fixed costs and variable costs increasing slower in proportion to productivity (Fig. 3).

**Fig. 4:**
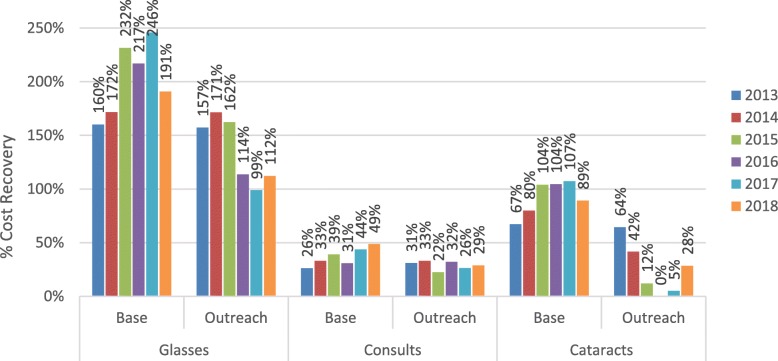
Cost recovery by activity, location, and year

Cost recovery differed significantly in the hospital versus outreach settings (Fig. 4). Of the consultations and operations performed at the base hospital, the 6-year average recovery rate was 37 and 92% for consultations and operations respectively. The comparable rates at outreach were 29 and 30% respectively, indicating that the eye program was operating at a more self-sustained level at the base hospital but relying on external funds for their outreach activities.

**Fig. 5:**
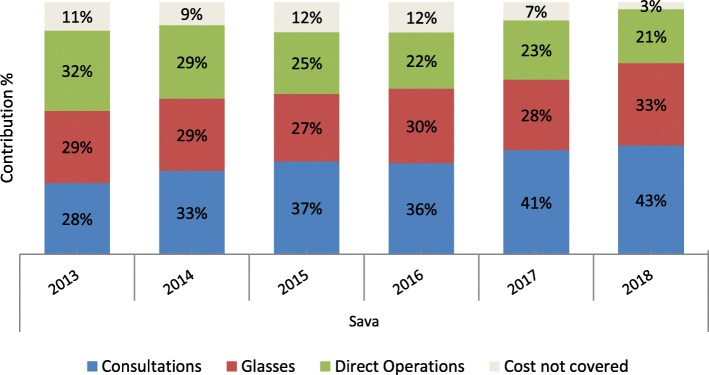
Patient revenue as a proportion of overall program cost

Sava covered 97% of their overall costs through patient revenue (Fig. 5). Consultations were the leading revenue source from patients followed by glasses and cataract operations. Donations and grants remained consistently low as did other revenue sources such as fundraising. Revenue from consultations and glasses grew steadily (Fig. 4). Surgical outreach was not conducted in 2016, however this did not significantly impact revenues, as these patients typically receive surgery free of charge, nor expenses, as the cost savings were offset by cost increases elsewhere. The most significant improvement occurred in 2017 when the program increased consultation fees from $8 to $11 USD. As a result, consultations increased contributions to overall cost recovery from 36 to 41% (Fig. 5).

In 2018, fixed costs accounted for 69% of total costs with staff salaries and bonuses accounting for approximately one-third of costs. The largest proportion of fixed costs was found in consultations (70%) and reflect the number of out-patients. The proportion of fixed costs attributed to cataract was 22%. The majority of variable costs consisted of medicine, cataract surgical consumables (such as intra-ocular lenses) and frames and lenses for the optical shop.

## Discussion

The program manager and ophthalmologist used the TDABC methodology to break down activities into time spent and resources used and to identify the key modifiable variables that influenced productivity and net profit. In keeping with this low-income setting and the institutions’ first efforts at financial management and economic modelling, the TDABC models they produced were limited compared to high-income settings. That is, while the Sava program was measuring and modelling the ophthalmologist’s activities for the first time, TDABC models of surgical [7] or diagnostic activities [8] in an industrialized setting such as Canada include multiple personnel, with time charts and sequence analysis.

Nevertheless, although the TDABC eye care models in the Sava eye unit were relatively simplistic compared to large complex hospital programs, the eye unit manager and ophthalmologist used them to effectively meet the challenges of more than 200% increase in eye care costs over the 5 year study period. In particular, they worked together to optimise clinical productivity, particularly time management of the ophthalmologist. This modified the uncontrollable cost increases (consumables, equipment and salaries) and allowed the investment in the optical shop and sale of glasses to provide sufficient revenue to cover program costs.

The Sava eye program clearly recognized that price could affect coverage. Nevertheless, it was necessary for program survival that they increase prices for consultation, for all patients, and for cataract, for people who could afford to pay. As shown in Table 1, the increased price did not significantly impact volume of consultations, glasses or cataract from year to year. More free surgery and more outreach would have helped the program to achieve full population coverage, but that was not sustainable in this setting. A donor-funded cataract surgery campaign (reduced price and supplemental support for outreach) was available in 2013 and it did result in the high cataract volume in that year.

The cataract operation TDABC model was the most complex and proved the most valuable in determining cost drivers, improving efficiency and increasing patient revenue [9]. Information from the model helped Sava increase fees from patients who came directly to the hospital (as opposed to those referred from outreach camps and had free surgery) in excess of the cost of their cataract operations. Reaching their overall (hospital and outreach) cataract break-even point was possible because these ‘direct’ patients subsidised those referred from outreach camps. In recognizing the importance of and achieving the ‘break even’ volume for cataract surgery, the Sava program was able to increase patient revenue to meet steadily increasing costs of equipment, personnel and supplies.

Techniques and addition of surgical assistants reduced the time taken for cataract surgery throughout this program. Greater efficiency meant greater productivity per time unit. However, the increased profit (more revenue per unit due to decreased time costs) was not reflected in reduced price to patients. The price needed to be maintained and, in fact, increased to meet the increasing costs of equipment and consumables.

Cataract cost containment was greatly facilitated by collaboration with KCCO, an external partner with access to foreign currency. It allowed savings by group purchasing of consumable items (intra-ocular lenses, needles, and medicines) in US dollars and avoided additional 15% annual inflation costs of the Malagasy currency.

For sale of glasses, the TDABC model provided useful but relatively self-contained (within the eye program) information in terms of personnel, supplies, costs and revenue. While recognized as essential to overall hospital profit; optical shop management involved a separate type of time management for shop staff, specific expertise in purchase and supply of frames and lenses, and patient (client) experience. The overall eye program challenge was ensuring that a high proportion of patients prescribed glasses by the hospital ophthalmologist actually purchased glasses in the hospital optical shop.

The optical personnel learned to conduct market assessments and develop marketing strategies and materials. Most of the new marketing strategies focussed on the optical shops, recognizing the need for substantial improvement in the physical space, the patient’s experience as a customer, and the range of frames and lenses available. Staff began adopting specific sales techniques to increase the proportion of customers purchasing glasses and selecting higher priced frames. Staff also began, albeit informally, to solicit customer feedback on the reasons for and against purchase decisions. In fact, the ophthalmologist came to recognize that a key factor influencing glasses sales from the hospital optical shop was whether he recommended the shop and if he actually escorted the patient to the shop following consultation. While this activity by the ophthalmologist increased the ‘cost’ of glasses (more ophthalmologist’s time) the TDABC model was not adjusted to account for this relatively small change in ophthalmologist role.

The Sava TDABC model provided the least value in program planning around patient consultation. Costs were directly dependent on the time taken by the ophthalmologist and revenue depended on a single consultation fee, which almost all patients were able to pay. Some efficiency was achieved by involving allied personnel to do measurement of visual acuity or refraction, thereby freeing some of the ophthalmologists’ time to see additional patients. The staff increased focus on waiting times, patient experience and satisfaction both to increase comfort and decrease waiting times.

Sava recognized that improved productivity and profit could not come at the cost of patient safety or quality of care and added much more rigorous monitoring of cataract surgical quality and began assessing patient and optical shop customer satisfaction, albeit informally.

Sava also recognized that, despite the relatively high cost of and low revenue generation from outreach services, providing these services in rural areas was an essential component of their Vision 2020 Program commitment. Sava did seek efficiencies through minimizing outreach fixed costs by having fewer, larger camps that lasted fewer days. However, the cost to the programs of finding patients through outreach was compounded as most clinical staff are away from the eye unit in order to provide outreach services.

The Sava program, while achieving self-sufficiency in terms of service delivery cost, did not achieve self-sufficiency in terms purchasing new or replacing ophthalmic or anaesthetic equipment. These additional costs, estimated as much as 15–20% of operating costs per year, required continued donations from external sources such as Seva Canada.

## Conclusion

Time-Driven Activity Based Costing methodology and its associated use in strategic decision-making became a useful tool to improve financial self-sustainability for a private eye care program in Sava, Madagascar.

## Data Availability

The datasets used and/or analysed during the current study are available from the corresponding author on reasonable request.

## Abbreviations

SALFA: Sampan’asa Loterana Momba Ny Fahasalamana
TDABC: Time-Driven Activity Based Costing
KCCO: Kilimanjaro Center for Community Ophthalmology
